# Effect of copper-impregnated linens on multidrug-resistant organism acquisition and *Clostridium difficile* infection at a long-term acute-care hospital

**DOI:** 10.1017/ice.2018.196

**Published:** 2018-09-20

**Authors:** Gregory R. Madden, Brenda E. Heon, Costi D. Sifri

**Affiliations:** 1 Division of Infectious Diseases and International Health, Department of Medicine, University of Virginia Health System, Charlottesville, Virginia,; 2 University of Virginia Transitional Care Hospital, University of Virginia Health System, Charlottesville, Virginia; 3 Office of Hospital Epidemiology/Infection Prevention and Control, University of Virginia Health System, Charlottesville, Virginia

## Abstract

Copper-impregnated surfaces and linens have been shown to reduce infections and multidrug-resistant organism (MDRO) acquisition in healthcare settings. However, retrospective analyses of copper linen deployment at a 40-bed long-term acute-care hospital demonstrated no significant reduction in incidences of healthcare facility-onset *Clostridium difficile* infection or MDRO acquisition.

Hospital room occupation by patients carrying Clostridium difficile or a multidrug-resistant organism (MDRO) impart increased risk of acquisition by subsequent patients and likely serves as a reservoir for nosocomial pathogens.^1,2^

“No touch” measures, such as hydrogen peroxide vapor or ultraviolet light for enhanced terminal room cleaning have been shown to improve disinfection and reduce healthcare-associated infections (HAIs); however, these strategies are time and labor intensive and can only be employed in unoccupied rooms.^3^ Constitutively active antimicrobial surfaces and fabrics represent novel approaches to reducing patient-to-patient transmission because they continuously act to reduce the bacterial burden.^3^

Copper has broad microbicidal activity including bacteria, fungi, and viruses, in addition to the vegetative and, with reduced potency, spore forms of C. *difficile*.^2,3^ Deployment of copper surfaces has been shown to reduce MDRO colonization of hospital surfaces.^2^ Limited clinical evidence also suggests that copper surfaces and/or linens may reduce HAIs among intensive care unit (ICU) patients (copper surfaces only),^4^ among non-ICU inpatients (surfaces and linens),^5^ and at a long-term care facility (linens only).^6^ We hypothesized that implementing copper linens in a long-term acute-care hospital (LTACH) would reduce incidence of healthcare facility-onset *Clostridium difficile* infection (HO-CDI) and MDRO acquisition.

## Methods

Copper-impregnated woven linens including bed sheets, fitted sheets, pillowcases, towels, and washcloths (Cupron Medical Textiles; Cupron, Richmond, VA) were deployed on October 6, 2014, at a 40-bed LTACH in Charlottesville, Virginia. The linens were removed over the month of January 2017 after monitoring for HO-CDI and MDRO acquisition showed no benefit.

We retrospectively analyzed healthcare facility-onset CDI (HO-CDI) events according to the National Health and Safety Network (NHSN) laboratory-identified (LabID) definitions^7^ and HO-MDRO acquisition. HO-MDRO acquisitions, defined as a new finding compared to known status, were detected by routine surveillance (perirectal or ostomy swab and MRSA nares swab on admission and weekly thereafter) or NHSN LabID HAIs.^7^ Swabs were tested for methicillin-resistant *Staphylococcus aureus* (MRSA) using Xpert MRSA polymerase chain reaction (PCR) assay (Cepheid, Sunnyvale, CA) and for vancomycin-resistant enterococci (VRE) using Spectra chromogenic agar (Thermo Fisher Scientific, Lenexa, KS).^8,9^ Carbapenem-resistant Enterobacteriaceae (CRE) and extensive drug-resistant *Acinetobacter baumannii* (≥3 antibiotic classes) were identified following selective incubation and subculture to Colorex agar (Northeast Laboratory Services, Win-slow, ME), followed by modified carbapenem inactivation with Carba-R PCR (Cepheid) or susceptibilities, respectively.^10–12^ Facility hand hygiene compliance auditing data were also collected during a combination of 12-hour day and night shifts.

Monthly incidence rates were analyzed over a 27-month control period prior to implementation (July 2012 through September 2014) followed by a 27-month intervention period (October 2014 through December 2016), and an additional 10-month control period (January 2017 through October 2017).

Rates of hygiene compliance (ie, hand hygiene before and after patient care) were measured through an anonymous auditing program that did not change over the course of the study period. An average of 40 hand hygiene opportunities were assessed per month. *Clostridium difficile* infection testing was performed using real-time PCR of the tcdB gene (Cepheid) during the entire study. Universal contact precautions were employed throughout the study period with good (>92%) audited compliance that did not vary significantly over time.

Statistical *P* values were obtained using 2-tailed independent samples *t* tests for equality of means. In addition, interrupted time-series analyses were performed using quasi-Poisson regression, with patient days as an offset and hand hygiene compliance as a covariate.

## Results

**Fig. 1.**
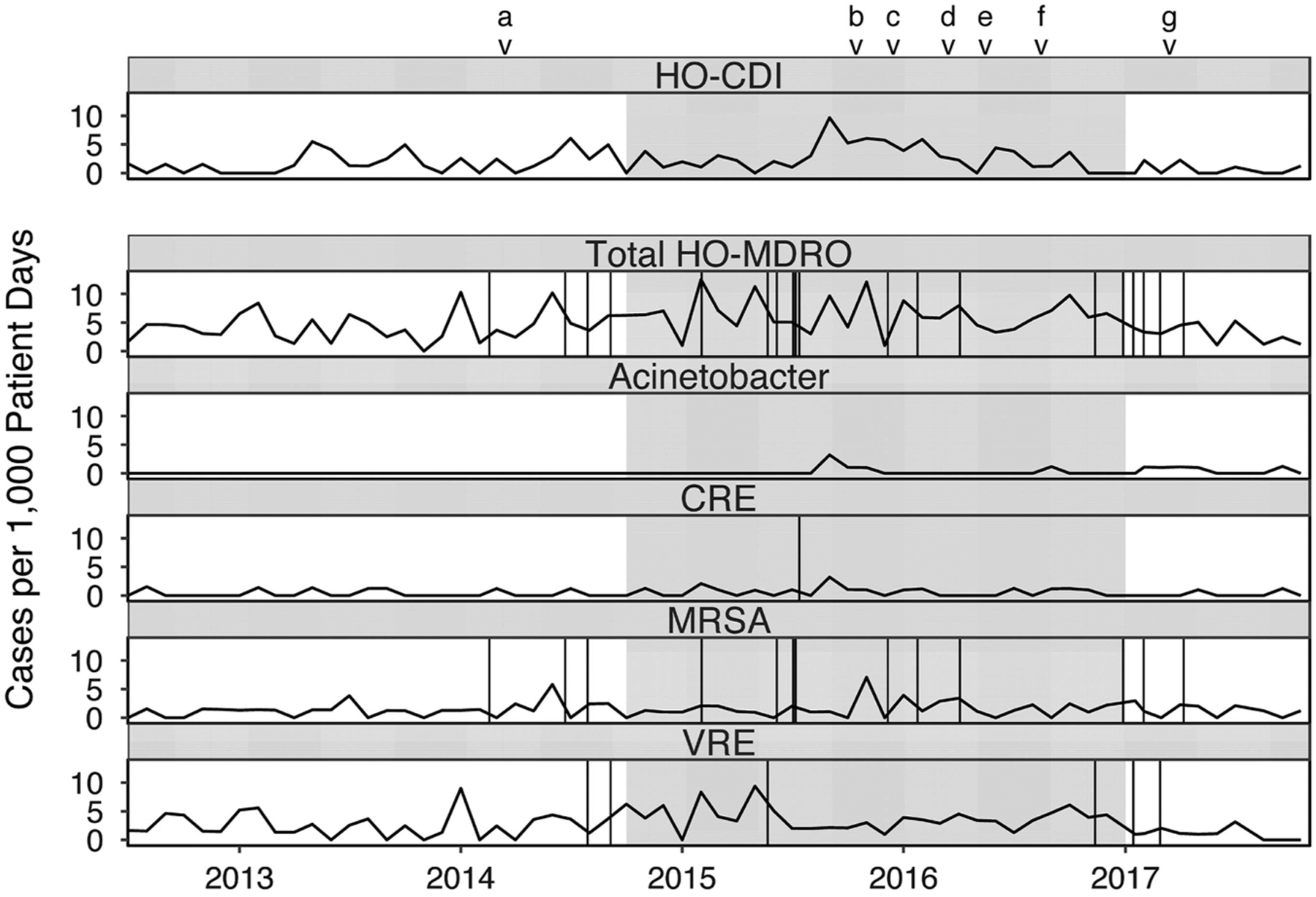
Rates of healthcare facility-onset *Clostridium difficile* infection (HO-CDI) and multidrug-resistant organism (HO-MDRO) acquisition events with copper linens intervention (grayed). Vertical lines represent NHSN-reported healthcare-associated infection events due to MDROs (CRE: urinary tract x1; MRSA: bloodstream x6, pneumonia x1, ear eyes nose throat x1, lower respiratory x2, skin and soft tissue x1, ventilator-associated event x2; VRE: bloodstream x1, gastrointestinal x1, urinary tract x4). Note. CRE, carbapenem-resistant Enterobacteriaceae; MRSA, methicillin-resistant *staphylococcus aureus*; VRE, vancomycin-resistant enterococci. ^a^Began offering yogurt to patients. ^b^Hand hygiene competency evaluation for all staff. ^c^10 new mattresses purchased. ^d^Larger *C. difficile* signs placed in rooms. ^e^Instituted twice weekly universal bleach disinfection of all rooms. ^f^Beds replaced due to mechanical failure. ^g^Bed deck covers deployed.

Overall, 29,342 patient days were observed during the control period and 25,243 patient days were observed during the intervention (Table 1). Copper linens were associated with significantly higher rates of HO-CDI (*P* = .023) and total HO-MDRO acquisition (*P* = .001). In subgroup analysis, differences in total HO-MDRO acquisition were largely attributable to VRE (2.1 acquisitions per 1,000 patient days during control vs 3.8 during the intervention; *P* = .002) and to CRE (0.3 acquisitions per 1,000 patient days during control vs 0.7 during the intervention; *P* = .044). Comparisons of the relatively small number of HO-MDRO infections during each period revealed no significant differences (*P* = .313). Rates of HO-CDI, HO-MDRO acquisitions, and changes in infection control–related practices over the study period are depicted in Fig. 1.

Poorer mean monthly hand hygiene compliance occurred during the intervention period (90.9% vs 95.3% for the control period). Quasi-Poisson models demonstrated a similar effect on total HO-MDRO acquisition (*P* = .001); however, HO-CDI was no longer significantly different among groups (*P* = .081). See the supplementary material for hand hygiene and modeling data.

## Discussion

Contrary to our hypothesis, our data do not suggest a beneficial effect of bed/bath copper linens for reducing HO-CDI or HO-MDRO acquisition. Our findings contrast with a study of Israeli long-term care facility patients that demonstrated significant reductions in HAIs (24% reduction), fevers (47% reduction), and bacterial loads measured from sheets with copper linens.^6^ Several major differences may explain our disparate findings, including the additional use of copper-impregnated personnel uniforms, high baseline HAI rates that could amplify copper effects, and differences in primary outcomes between the studies.^6^

Although our data demonstrated increased HO-CDI and HO-MDRO acquisition during the intervention period, there are several reasons to question a causative role by copper, including the well-described reduced bioburden by copper materials^2,3^ and studies showing clinical benefit in other settings.^4–6^ The likelihood of the development of microbial resistance to copper is likely small due to the multiple mechanisms of copper’s microbicidal action. In the United Kingdom, bacterial resistance has not been observed, despite wide deployment of copper.^2^ However, staff and patients were not blinded to the intervention; thus, we cannot rule out that copper linens may have resulted in degradation of other infection control practices, including hand hygiene, possibly leading to increased pathogen transmission. Similar hypotheses have been advanced to explain the negative correlations between glove use and hand hygiene compliance.^13^ Notably, no changes to the institutional antibiotic formulary or policies occurred during the study period.

This study has several limitations. While quasi-experimental studies are often pragmatic within the field of healthcare epidemiology, time-varying factors and overlapping interventions (Fig. 1) may have introduced bias by influencing surveillance detection of infection events or MDRO transmission. Also, the NHSN surveillance definition for CDI changed beginning in January 2016 from infection surveillance reporting to LabID event reporting, so that symptoms of CDI were no longer required to be present.^7^ There-fore, CDI detection may have been inflated during the latter portions of the intervention and control periods. Linens were laundered using standard protocols (according to manufacturer recommendations) and although the manufacturer reports the linens degrade microbes for the life of the product, we cannot rule out the possibility that the antimicrobial properties may have waned over time.

Despite some limitations, our study has several notable aspects. First, the add-and-remove treatment design strengthens the evaluation of time-varying factors.^14^ Second, point-prevalence MDRO surveys were collected on admission and weekly to determine new colonization events. Most previous studies of copper-containing products only reported rates of clinical infections but not rates of new colonization events; predictably, this will underestimate the rate of new total acquisitions.

While we did not observe a beneficial effect, copper-impregnated surfaces with or without copper-impregnated linens may be useful in other settings as an adjunct strategy to existing infection control practices, particularly if incidences of HAIs and MDRO transmission are high and infection control practices do not degrade with their use. Trials with more rigorous study designs (eg, randomized clinical trials) and relevant endpoints (eg, new total acquisition rates) are needed to define the efficacy of copper linens and/or surfaces in healthcare settings.

## Supplementary Material

Sup001

Sup002

Sup003

## Figures and Tables

**Table 1.** Effect of Copper Linens on Rates of HO-CDI and HO-MDRO Acquisition

Infection Type	Control Period Total Events/Patient Days, Incidence Rate (%)^a^	Intervention Period Total Events/Patient Days, Incidence Rate (%)^a^	*P* Value
HO-CDI	44/29,342 (1.5)	70/25,243 (2.8)	**.023**
HO-MDRO acquisition	115/29,342 (3.9)	160/25,243 (6.3)	**.001**
MRSA	39/29,342 (1.3)	40/25,243 (1.6)	.496
VRE	62/29,342 (2.1)	97/25,243 (3.8)	**.002**
CRE	9/29,342 (0.3)	17/25,243 (0.7)	**.044**
*Acinetobacter*	5/29,342 (0.2)	6/25,243 (0.2)	.540
All MDRO HAIs	9/29,342 (0.3)	11/25,243 (0.4)	.313
MRSA	5/29,342 (0.2)	8/25,243 (0.3)	
VRE	4/29,342 (0.1)	2/25,243 (0.1)	
CRE	0/29,342 (0.0)	1/25,243 (0.04)	
*Acinetobacter*	0/29,342 (0.0)	0/25,243 (0.0)	

Note. HO-CDI, healthcare facility-onset *Clostridium difficile* infection; HO-MDRO, healthcare facility-onset multidrug-resistant organism; MRSA, methicillin-resistant *Staphylococcus aureus*; HAIs, healthcare-associated infections; VRE, vancomycin-resistant enterococci; carbapenem-resistant Enterobacteriaceae.

a Incidence rates are per 1,000 patient days. Significant *P* values < .05 are shown in boldface type.
