# Supplementary figures and images for: Effect of copper-impregnated linens on multidrug-resistant organism acquisition and Clostridium difficile infection at a long-term acute-care hospital
Source: Infect Control Hosp Epidemiol. Author manuscript; Available in PMC 2020 Mar 10. (PMC7063582; doi:10.1017/ice.2018.196)

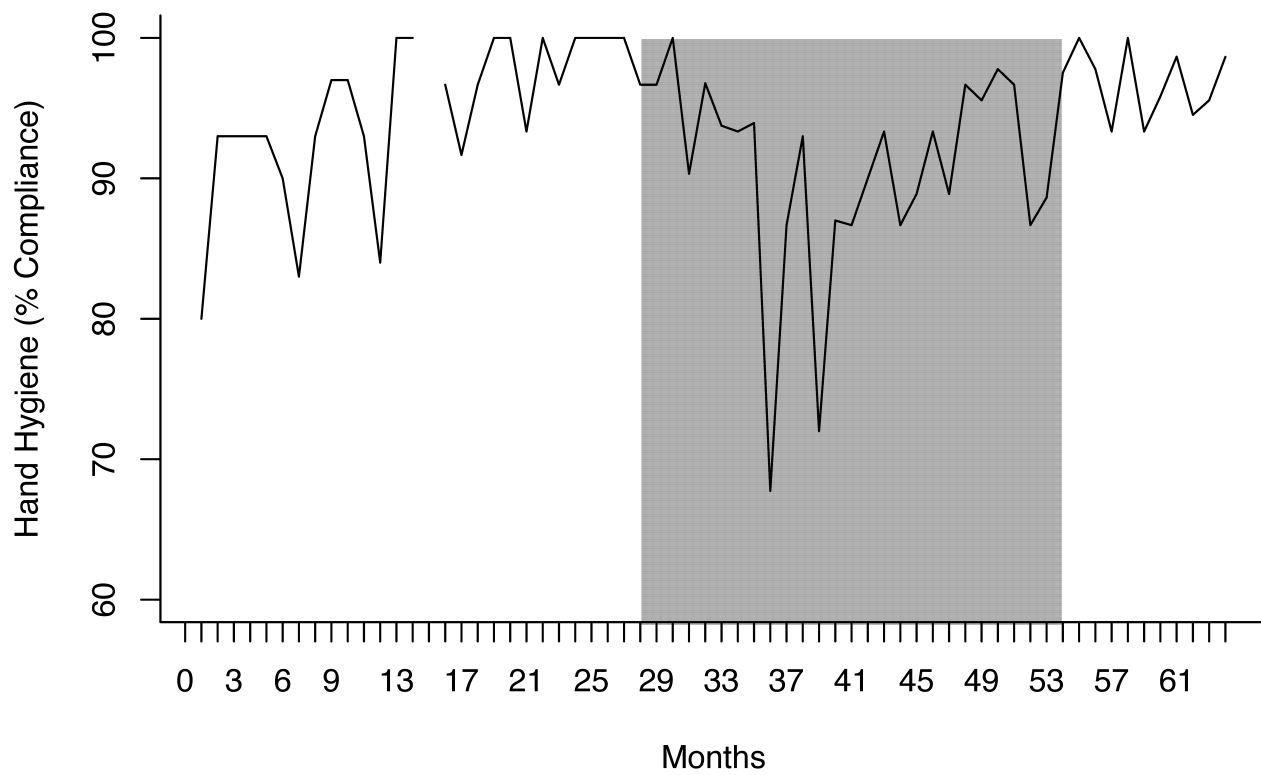

Supplement: Sup001 [file NIHMS1553858-supplement-1.pdf]

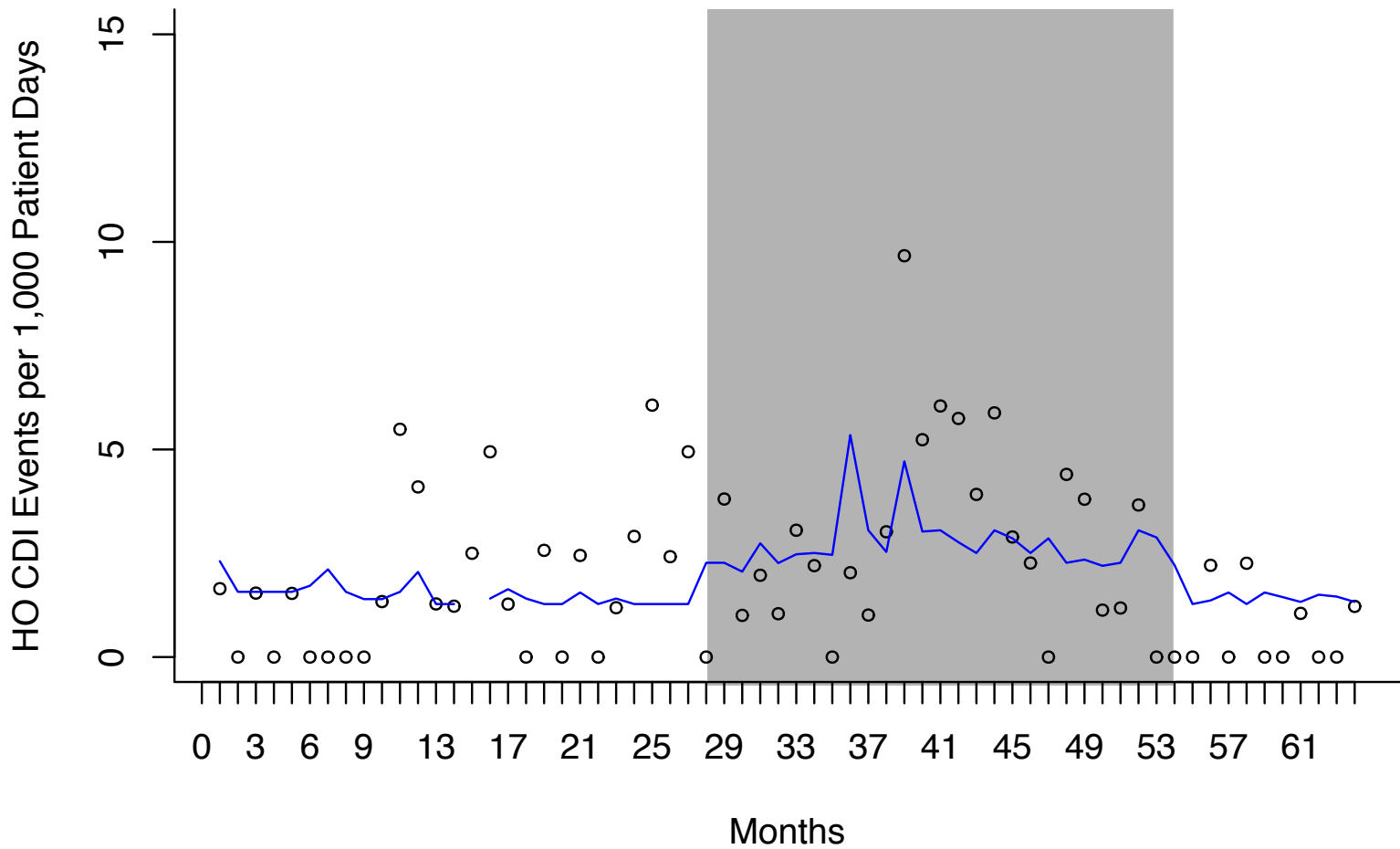

Supplement: Sup002 [file NIHMS1553858-supplement-2.pdf]

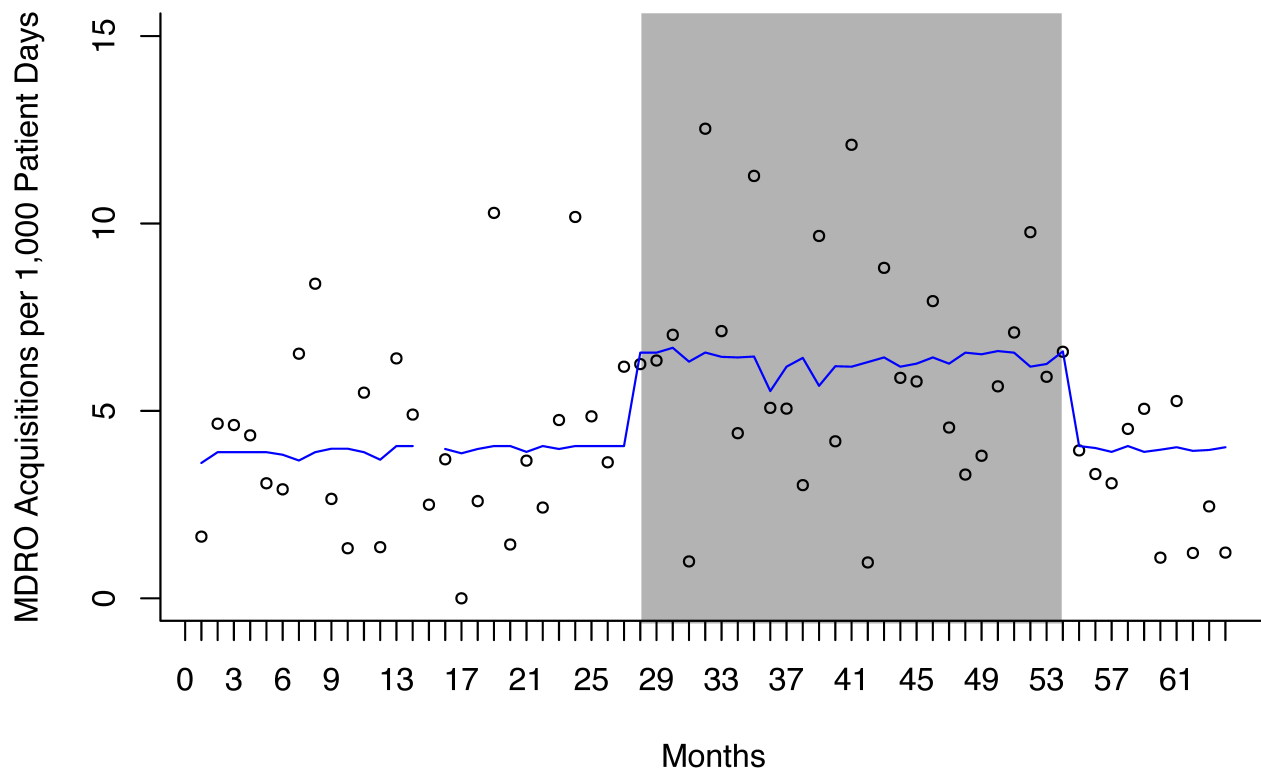

Supplement: Sup003 [file NIHMS1553858-supplement-3.pdf]
